# Philosophers in research ethics committees—what do they think they’re doing? An empirical-ethical analysis

**DOI:** 10.1007/s11019-021-10044-1

**Published:** 2021-08-16

**Authors:** Charlotte Gauckler

**Affiliations:** University of Greifswald, Baderstraße 6-7, 17489 Greifswald, Germany

**Keywords:** Ethics committees, Ethics expertise, Medical ethics, Applied ethics, Relationship between medical ethics and healthcare law

## Abstract

Research ethics committees in Germany usually don’t have philosophers as members and if so, only contingently, not provided for by statute. This is interesting from a philosophical perspective, assuming that ethics is a discipline of philosophy. It prompts the question what role philosophers play in those committees they can be found in. Eight qualitative semi-structured interviews were conducted to explore the self-perception of philosophers regarding their contribution to research ethics committees. The results show that the participants generally don’t view themselves as ethics experts. They are rather unanimous on the competencies they think they contribute to the committee but not as to whether those are philosophical competencies or applied ethical ones. In some cases they don’t see a big difference between their role and the role of the jurist member. In the discussion section of this paper I bring up three topics, prompted by the interviews, that need to be addressed: (1) I argue that the interviewees’ unwillingness to call themselves ethics experts might have to do with a too narrow understanding of *ethics expertise*. (2) I argue that the disagreement among the interviewees concerning the *relationship between moral philosophy and applied ethics* might be explained on a theoretical or on a practical level. (3) I argue that there is some lack of clarity concerning the *relationship between ethics and law* in research ethics committees and that further work needs to be done here. All three topics, I conclude, need further investigation.

## Introduction

During the last decades, the importance of ethics committees has been continuously rising in industrial nations. This rise can be seen as a reaction to the rapid increase of new fields of action calling for novel decisions due to technological progress. Together with an increasing moral and phenomenological pluralism, due not least to globalisation, these developments lead to a new need for orientation and to a “call for ethics” (Kaminsky 2005). We may ask, however, whether ethics committees are successful in answering this call by actually providing the called-for ethics—and if so, what kind of understanding of ethics their work is based on. The fact that these committees have ethics in their name suggests that the discussion of ethical problems is at least part of their job. What stands out from a philosophical point of view is the fact that there are hardly any philosophers to be found in most ethics committees. Assuming that ethics is primarily a philosophical discipline, this substantial but not total absence of philosophers—or of any kind of “ethics experts” for that matter—in ethics committees is surprising and raises several questions: What is the role of ethics experts in ethics committees in general and of philosophers in particular? Considering that, especially in clinical ethics committees, there are often experts on medical ethics who don’t necessarily have a philosophical background, what is the difference between medical ethics and philosophical ethics? Should there be more philosophers in ethics committees or are the few that are there completely expendable?

Of course, the answers to these questions may be different for different kinds of ethics committees. Concerning Germany, it makes sense to differentiate between three kinds of ethics committees: ethics councils, like the *Deutscher Ethikrat* whose purpose is to offer political counsel about substantial matters; health care ethics committees (HCECs), which give council in concrete health care situations; and research ethics committees (RECs),^1^ which counsel researchers but also essentially decide whether the designs of clinical studies are ethically sound or not. In Germany, (medical) research ethics committees are especially well established, not least because their consultation is statutory for all research including human participants, material and data. These RECs are organised in the *Arbeitskreis medizinischer Ethik-Kommissionen*, they have established more or less uniform procedures and the composition of their members is partly standardised. They consist mostly of medical professionals, and they all need to have at least one member with legal training by law and usually a person with experience in the field of ethics. Only some statutes also call for a philosopher or a person from the humanities instead. Very few statutes call for a layperson or a patient representative as well. This relative uniformity of RECs makes them a good subject for my study. Moreover, they are also a very interesting subject due to the fact that the role of philosophers in these committees is especially unclear. The function of RECs, more than that of the other sorts of committees, is an administrative one. They have to assess whether a medical study meets the ethical standards that have been partly established beforehand and then decide on the basis of laws whether the study may be conducted. So because they are an administrative institution that has ethics in its name, it is unclear to what extent their normative framework comes from ethics and to what extent it comes from law. And since the role of ethics in the committee is unclear, so is the role of the ethicist. In German philosophical discourse, only few authors have addressed the role of the philosopher in research ethics committees (Birnbacher 2002; Gesang 2002; Siep 2002) and no empirical studies have been conducted on this topic.

Against this background, the aim of my qualitative interview study was to find out what those few philosophers in RECs think about their role in the committee, whether they see themselves as ethics experts, the influence of their philosophical background on their role, how they perceive the other members’ expectations of them and what they think of the committee and its function in general.

## Methods

I consider this study an empirical-ethical one insofar as the the empirical part was prompted by ethical consideration and its findings in turn inform my ethical research. The ethical analysis of the results of the empirical study will take place in part in the discussion section of this paper, but will further inform my own and hopefully others’ future ethical research on the proper role of philosophers in ethics committees, by pointing out new questions that need to be explored and providing information on the context of RECs (see Musschenga 2005).

The empirical study is based on eight semi-structured expert interviews with philosophers in research ethics committees. Of these eight participants, six present as male and two as female, seven are current or former members of a REC and two are current or former members of the German ethics committee for stem cell research, *Zentrale Ethik-Kommission für Stammzellforschung* (ZES). Some of the participants also have experience working in other types of ethics committees, such as HCECs, which they were also asked about but which was of no significance for recruitment. In order to find suitable participants for the study, the membership lists of all RECs of German universities and medical associations were searched. RECs were chosen as the main subject of the study in part due to their being well established and therefore numerous, and to the concomitant comparability to each other.

The criterion for inclusion, besides the current or former membership in a research ethics committee, was a degree in philosophy; the exclusion criterion was a degree in medicine or a different kind of education that could be seen as sufficient qualification for membership in the committee. In this way it was ensured that only those members were questioned who are actually in the committee as philosophers and not for other reasons. Additionally, it was hoped that in this way distinguishing criteria between philosophers and other experts in medical ethics, for example those with a medical background, could be found.

In addition to the search in membership lists, former members of research ethics committees were found through references by those contacted and by others. Of the approximately ten eligible philosophers who were found this way, seven agreed to participate in the study. Since one of those seven was a current or former^2^ member of the ZES, another current or former member of the ZES was recruited in order to potentially be able to gather helpful information on the differences and similarities between these two types of ethics committees.

Ahead of the interview, the participants received written and oral information about data protection and possibilities of withdrawal from the study, and gave their written consent. The interviews were recorded in audio and pseudonymised in the process of transcription. In doing so, all personal data was deleted, as well as details about the respective ethics committee, the participants’ gender and all other information that could give any indication of the participants’ identity.

The interviews were conducted as semi-structured guided expert interviews (Lamnek 2005; Galletta 2013; Bogner et al. 2014; Adams 2015) in the fall of 2018. The interviewees were asked about the general modus operandi of the committee, the topics that are discussed in the committee, their own role in the committee, the influence their philosophical identity has on their work in the committee and how content they are with the workings of the committee. The duration of the interviews varied between approximately 40 and 60 min.

The transcripts were then subjected to a qualitative content analysis (Mayring 2000; 2004; 2008; 2014). Four categories were defined based on the questionnaire: 1. ‘self-concept’, 2. ‘role-ascription through other members’, 3. ‘function and modus operandi of the committee’ and 4. ‘satisfaction with the committee and own role’. For this paper I only look at the results from the first category, ‘self-concept’.

The relevant passages of the transcripts were then preliminarily assigned to the categories, paraphrased and partially re-assigned more precisely. The paraphrases thus obtained were then individually generalised within their respective categories and reduced according to the rules of summary outlined by Mayring (2000; 2008; 2014). From this material, six sub-categories were defined within the category ‘self-concept’ and the reductions were assigned to the categories: ‘Ethics Expertise’, ‘Specific Competencies’, ‘Medical Layperson’, ‘Medical Ethics versus Philosophy’, ‘Ethics and Law’ and ‘Normative Framework’. Thereafter followed the comprehensive summary for each sub-category.

The language of the interviews was German, the transcripts are in German language and all quotes were translated by the author.

## Results

### Ethics expertise

It is generally agreed among the interviewees that philosophers aren’t ethics experts in the sense that they are in a position to tell other members of the committee what is right and what is wrong or how to solve an ethical issue:

**P 6**

Whereas naturally you can’t, let’s say, as a professional ethicist, just bring the solution to the table and say: ‘Now I’m going to tell you how we solve this problem’. Because, it is in the nature of things that many of these […] ethical questions demand an ethical discussion […] without one having a right or wrong answer.

### Specific competencies

However, there is less agreement to be found on whether philosophers are ethics experts in a different sense. The interviewees generally talk very little about philosophical or ethical expertise directly, and when they do, there is always some uncertainty: “Well, whether it is the philosophical expertise … difficult.” (P 6) As a matter of fact, it is striking that in spite of repeated mention of terms like “expert”, “expertise” and the like by the interviewer, the interviewees hardly take up that choice of words at all. Instead, there’s more talk about certain competencies that a philosopher can contribute to the committee. Whether these competencies are specifically philosophical or there are other professions, e.g. theology or humanities in general, which could contribute these competencies, is one contentious point. However, it is generally agreed that if specifically philosophical or ethical competencies are applicable, this isn’t the case “in day-to-day business” (P 7), but rather in special circumstances—for example when unprecedented questions arise. Knowledge of ethical principles, debates and publications is predominantly mentioned as a specific philosophical or ethical^3^ competency, however, this is also partly relativised. P 8, for example, states that the knowledge of ethical principles isn’t reserved for the philosophical member of the committee:

**P 8**

Whereas the others of course also know these principles. That is to say the other members of the committee are essentially also familiar with the canon (basic regulations) in research ethics.

Another competency that is perceived as specifically philosophical or ethical is the detection of “imbalances in the […] discourse” (P 1) which in turn is associated with the knowledge of the debates: the philosopher has the ability to note when the discussion in the committee is, for example, too fixated on a certain value and thereby loses sight of the counterside. And this the philosopher does by referencing the (academic) debate on the topic in question:

**P 1**

This, I would say, is actually the thing that is genuinely philosophical […], saying: ‘there is a tension that needs constant management and we are currently tilting’; keeping this in mind and saying: ‘this is always a big debate and in this debate each concept has an antagonist’.

Apart from this, among the most common competencies that are named as philosophical ones are the systematisation of positions and intuitions, categorisation and typification, as well as the testing of texts for clarity, stringency and coherence and the detection of suggestive phrasing. Interviewees also name topics that the philosopher can contribute to, namely: vulnerable patients, risk–benefit assessment, informed consent and topics they have published on. Accordingly, interviewees are fairly agreed that the philosopher is particularly responsible for reading the patient information—on the one hand because this is where topics like informed consent and patient autonomy, as well as the detection of suggestive phrasing, are especially relevant. On the other hand, the reading of the patient information is seen as a strategy in light of difficulties in understanding: As a medical layperson the philosopher can most easily get an idea of the respective study by reading information that was specifically written for laypeople—considering that patients commonly aren’t medical professionals. In her role as medical layperson the philosopher can also assess the intelligibility of the patient information—unlike, or at least better than, the medical members of the committee.

### Medical layperson

Some interviewees deem the role as layperson as the central role, or one of the central roles, of the philosopher in the research ethics committee. For example, one of the interviewees states that there is no demand for any specifically philosophical competencies in the committee, yet it is important that there are non-medical members involved in discussions, “because physicians sometimes see problems too unilaterally as technical, hence often don’t think of fundamental questions, [and the non-physician] takes on the patient’s perspective” (P 2). P 2 also states that for this capacity of the non-medical member of the committee, philosophers, other humanities scholars and legal experts are all equally qualified, since what matters is mostly the capacity for subtle differentiation of terms with distinct semantic contents.

### Medical ethics versus philosophy

Another interviewee (P 7), however, considers the task of philosophers in research ethics committees to be twofold: On the one hand they are medical laypeople—and in this capacity they could be replaced by anybody because it is simply a matter of taking the perspective of the patients:

**P 7**

And this can also be contributed by a theoretical philosopher, yes, it can be contributed by a theologian, it can be contributed by a woman on the street, this perspective.

On the other hand, according to P 7, philosophers are also in the committee as medical ethicists. Yet P 7 doesn’t understand the competencies in medical ethics to be the same as philosophical competencies—the crucial prerequisite being, not a degree in philosophy, but knowledge of medical ethics, guidelines and the like:

**P 7**

[…] of course, one also contributes one’s medical ethical knowledge, but that’s not philosophical knowledge, that’s medical ethical knowledge, so I don’t start by arguing with Kant, but instead with, well, non-instrumentalisation as an ethical guideline or the like, next to other ethical guidelines.

All in all, the interviews suggest that the ethical member of the committee benefits from knowledge that is additional to their degree in philosophy, particularly medical ethical and research ethical knowledge. Moreover this additional knowledge is in some cases seen as a helpful additional qualification, whereas the philosophical knowledge would already be sufficient for work in the committee:

**P 8**

Well, the precise reading of texts and arguments and abstract interrelationships alone is of course something one learns when studying philosophy, and therefore, I think, everyone who has a degree in philosophy would be generally qualified to work in such a committee. But I, personally, also have a focus of work in research ethics […]. Therefore, I think I am especially qualified for this kind of work.

Others however, in line with P 7 above, consider the additional knowledge essential:

**P 5**

I think one must have a certain educational background [in medicine], but I don’t think it is necessary to study medicine. There are, after all, several courses of study nowadays that impart professional knowledge in medical ethics and it has also become more common to work together with medical colleagues […].

In fact, P 7 even holds that one doesn’t need much philosophical knowledge or even a degree in philosophy at all in order to take on the role of the ethicist in a research ethics committee:

**P 7**

Well, a little bit of Kant may come in through the backdoor, but that little bit of Kant you can do if you have taken part in an online postgraduate program on medical ethics.

So while P 7 seems to think that philosophical knowledge is at best a part of medical ethics knowledge, others seem to consider applied ethics knowledge, of which medical ethics knowledge can be counted as part, philosophical knowledge:

**P 4**

[…] among humanities scholars, philosophers and theologians are of course most suitable. That is, as regards the patient information as a type of text, a German philology or literary scholar would be equally suitable. But when it comes to questions of autonomy, to harm-benefit-questions … […] If one doesn’t have at least a little applied ethics in the back of one’s mind, one doesn’t really see these problems.

What is striking is that among the interviewees the ones who stress the difference between medical ethics and philosophy and underline the importance of medical ethical knowledge for work in the committee, or who see themselves more as medical ethicists, are generally the ones who work in the institutional context of medical ethics, i.e. departments of medical ethics. The ones with a more traditional philosophical career, professors of philosophy, tend to make no distinction between philosophy and medical ethics as disciplines.

### Ethics and law

Another topic that arose in the interviews is that of the relationship between ethics and law or between ethical and legal regulation, and also the differences in function between the ethicist and the legal expert. While P 1 states that the ethical debate generally takes place and is contributed to by all members after the legal questions have been settled:

**P 1**

It was about vulnerable groups and the question to what extent they could be included in certain things. So, the legal part was resolved and then there was some ethics on top: and they have to be protected specifically.

Others see these two domains as less separated. In fact, P 2 sees a “considerable congruence” between the tasks of the philosopher and the legal expert. It is not just that both of their main tasks are according to P 2 the subsumption of particular cases under general principles.

**P 2**

Everyone knows the legal regulations, but how they are to be applied to the individual case, this task of subsumption that the legal expert has to do, this isn’t a mechanical process, it’s about following the purpose of the law. This is actually something that not only the legal expert does. In the same manner, we also have to apply general ethical principles to individual cases. And that is a procedure that is generally analogous to what the legal expert does.

On being asked whether they both use similar methods but in their respective domains, P 2 claims that no, ethical and legal questions are not generally separated in the committee. According to P 2 there is a plethora of questions that arise from legal regulations which leave room for ethical argument or questions that have not been legally regulated (yet) and in addressing these questions, there is no substantial difference between philosophers and legal experts.

The view that the main domain of philosophers or ethicists in research ethics committees is the interpretation of legal regulations, can also be found in P 4’s and P 5’s accounts of the work of the ZES: the usage of stem cells being so thoroughly regulated by law that there is no room for ethical considerations aside from the interpretation of the law. Yet both P 4 and P 5 state that this applies even more to the ZES than to the RECs. P 6 confirms this view to the extent that she or he too states that ethical questions mostly arise from areas where the legal regulations aren’t clear. Additionally, P 6 lists the knowledge of legal regulations as one of the competencies that ethicists contribute to the committee.

### Normative framework

When asked whether they have a specific normative framework for their work in the committee or whether they think that the ethical tradition they subscribe to is relevant for their work, the interviewees were again rather unanimous in stating that their own position does not matter; that, for example, a utilitarianist and a Kantian do the exact same thing in the committee:

**P 2**

[…] one’s own position hardly matters. Because the questions are so local that what is essentially required is rather general ethical power of judgement. And this is independent from, let’s say, the greater position one advocates. This is casuistry, it is a matter of the individual case. And there are differences there but they can hardly be tracked back to any underlying overarching ethical theories.

While some, like P 2, view the work in the committee as casuistry where general ethical power of judgement is required, others rather describe what happens in the committee as the application of principles or guiding principles to individual cases. Yet, as we have seen above, it is not clear whether this application of principles is something they have a special expertise for or whether anyone (who is a member of the committee) could do this. But they are rather unanimous in stating that the principles or guidelines are not developed in the committee but outside of it, for example that they derive from the Declaration of Helsinki or just from the general medical ethical discourse, or from certain committees with the authority to issue guidelines. Seldom do they see actual debates on fundamental issues happen in the committee.

### Summary of results

Looking at the results from the interviews, some similarities and some differences are conspicuous. First of all, although all of the interviewees are or were in some way or another a member of the committee on account of their “experience in the field of (medical) ethics”, there was general agreement among them that they are not ethics experts, at least not in the sense that they could tell the other members what is right and what is wrong. Agreement can also be seen in terms of the competencies that were mentioned as the ones that the philosopher/ethicist contributes to the committee: namely hermeneutical skills, argumentative skills, knowledge of debates and principles and the ability to “think outside the box” (P 6). What’s different is the interviewees’ assessment of whether these competencies are philosophical or (applied/medical) ethical competencies, or no special competencies at all. Another interesting topic that came up in the interviews is the relationship between ethics and law and how the role of the ethicist and the legal expert in the committee differ. It seems to be unclear whether ethical and legal considerations are being treated separately in the committee or whether they sort of merge in the interpretation of laws.

## Discussion

### Previous research to consider

To my knowledge, there has only been one recent qualitative study on the roles of members of RECs, by Janssens et al. (2020). This study asked members of a REC in the Netherlands about their roles and responsibilities in the committee, and identified five roles: protector, facilitator, educator, advisor and assessor. According to the authors, these roles may overlap in practice, but are helpful to keep in mind for analysis and to prevent single roles from being too dominant in the discussion. The results of my study show that at least four of the five roles can be found in the participants’ self-assessment: being primarily responsible for the patients’ information and informed consent puts them in the role of protector, not being stricter or even less strict than other members puts them in the role of facilitator, highlighting their specific competencies puts them in the role of advisor and the whole description of the process of reviewing studies, bringing their respective knowledge together and striving for consensus puts them in the role of assessor. Only the role of educator wasn’t visible in the interviews, because the focus was more on the dialogue within the committee and not so much on the one with the researcher. All in all, the interviews also confirm the result of the previous study, according to which the roles overlap and can’t be assigned to individual members.

### Strengths and limitations of the present study

Talking to philosophers about philosophy as a philosopher has certain qualities to it that can be seen both as strengths and as weaknesses. Unlike non-expert participants in interview studies, they cannot only talk about their experience but possibly have also reflected on them or are able to reflect on them at a high level. Unlike with other expert interviews, here the interviewer was also a philosopher and therefore may be seen by the participants more as a conversational partner on equal ground. This has the potential both to uplift the quality of the data, providing a high amount of insight into the interviewees’ perception, but also to distort facts about the processes due to too many layers of analysis. Additionally, the study’s aim of finding out how important philosophers are in research ethics committees certainly has the potential to cause defensiveness in the interviewees, triggered by feeling like the worth of their work is being questioned. This, again, could have distorted their answers. But another advantage of the interviewer also being a philosopher is that this might have given the interviews more of the character of self-reflection than of an inquiry from outside.

As with all qualitative research, the results can only show what is and not what isn’t. Nothing can be said about things that the participants did not mention. So even when the interviewees are unanimous in their answers this doesn’t mean that their answer is the correct or the only possible answer. The number of participants in the study was rather small, in part due to the fact that there aren’t many philosophers in research ethics committees, but in part also because there was no clear way to find all potential participants, especially former members of committees. It also lies in the nature of qualitative research that the results are strongly dependent on the researcher and can be distorted by biases both in the questions asked and in the interpretation of the answers. Even though a lot of care has gone into identifying and removing any built-in biases, the results are undoubtedly shaped by subjectivity to a certain extent.

So the data from the interviews cannot simply be generalised. But what it can do is prompt new questions and point towards directions where there is more theoretical work to be done. In my view it particularly opens up three questions or themes which I would like to elaborate on briefly. The first one is the question of ethics expertise. Are the interviewees really not experts or is there perhaps a more fruitful way of understanding the term? The second one is the question of where the skillset they share belongs. Does it belong to philosophy or to medical ethics, and what is the difference between the two? The third part of the discussion touches on the relationship between ethics and law in the context of RECs.

### Ethics expertise

The interviewees didn’t see themselves as ethics experts, but there also seemed to be a certain concept of ethics expertise underlying this assessment, namely one according to which an ethics expert is someone who knows what’s right or wrong, at least better than non-experts. Yet in practice the interviewees were all called into the committee on account of their knowledge or experience in the field of ethics in one way or another. This suggests that whoever called them into the committee might have attributed at least something similar to expertise to them, and also that third parties (other members of the committee or society in general) might have expectations that revolve around some concept of expertise relating to members of a committee who are in the committee on account of their academic knowledge. Furthermore, the interviewees also broadly agreed on the fact that they do have some competencies that they can contribute due to their background. So in order to manage expectations of what a person with a background in ethics can do, it might be helpful to look into alternative, broader concepts of ethics expertise.

Only very few authors have actually argued for the view that philosophers’ ethical judgments are truer or more likely to be true than those of non-philosophers (e.g. Singer 1972; Gordon 2014). Different reasons have been brought forth against the notion of ethics expertise. Some of them are conceptual reasons, like the claim that in order to be an ethics expert one needs to command special knowledge about moral facts, which isn’t possible either because there are no moral facts or because there is no way of knowing them or of knowing whether someone who claims to know them really does so (see for example Cholbi 2007; Iltis and Sheehan 2016). But there are also normative reasons brought forth as to why we shouldn’t assume that there is expertise in ethics. The idea that some people know better than others what is right or wrong, like philosopher kings or queens, is of course one with very problematic implications. David Archard (2011) argues that the idea of ethics expertise in the sense that a few experts decide what’s right and wrong runs counter to the democratic ideal that thrives on people’s capacity to govern themselves. Similarly, there has been discussion whether the idea of ethics experts or the practice of receiving ethics support threatens people’s autonomy (see for example Driver 2006; Rasmussen 2011).

But understanding ethics expertise in this autonomy-threatening way is not a given. There are other, more nuanced and less robust understandings of ethics expertise. I suggest to call the robust kind of expertise moral expertise, because it focuses on a substantial normative answer to a moral question. Assuming that ethics is to be understood as the methodological reflection on morality, ethics expertise, as I understand it, focuses more on the kind of knowledge that helps one come to a justified verdict on moral matters. This would include but might not be limited to the competencies that the interviewees named when asked what they contribute to the committee: hermeneutical skills, argumentative skills, knowledge of debates and principles and the ability to “think outside the box”. This understanding has also been present in the discussion on ethics expertise, but rarely has the distinction been made explicit by giving the two types of expertise different names.

One author who has also made this distinction is Lisa Rasmussen (2011), who developed a concept of ethics expertise for the context of clinical ethics consultation [the equivalent of what I have called health care ethics committee (HCEC)]. She identifies four ways in which ethics experts fare better than laypeople when it comes to making recommendations about moral questions without relying on knowledge about moral facts. According to Rasmussen, clinical ethics consultants are better than laypeople at:

1. Identifying clearly wrong answers
2. Reasoning “from a given moral premise to its implications, based on context”
3. Identifying “the full range of moral values and stakeholders involved in a situation”
4. Finding creative solutions to dilemmas

This expertise, according to Rasmussen, is based on “knowledge relying on the clinical context […], institutional policy, state and national law, norms of human behaviour […], and implications of moral premises and principles” (Rasmussen 2011).

And in fact, this does sound quite similar to the competencies that the interviewees mentioned as being particularly helpful in RECs: Being able to identify clearly wrong answers (1) and reasoning from a premise to its implications (2) comes with the ability to look for stringency and coherence. The ability to identify “the full range of moral values and stakeholders”(3) is similar to the detection of imbalances and maybe the role as medical layperson and patients’ advocate. Merely the fourth point, “finding creative solutions to dilemmas”, doesn’t correspond to anything the interviewees said. Maybe this is due to the fact that RECs have a smaller scope of decision-making due to their essentially being an administrative institution. There are also other differences between clinical ethics consultation and RECs that might result in different requirements for ethics expertise. First, ethics consultation is focused on the clinical context, while RECs are focused on research. So the knowledge of clinical context, institutional policy and norms both of law and of human behaviour that is required of the ethics expert would have a different focus. While a clinical ethics consultant (CEC) might need knowledge on medical treatment and laws concerning, say, euthanasia, an ethics expert in a REC rather needs knowledge on research methods and laws concerning medical research. Second, clinical ethics consultation is focused on an individual patient, and the aim is to find out their values and preferences, whereas RECs have to decide on and in line with general principles.

These differences, which I could only briefly sketch here, call for a more detailed investigation of what ethics expertise in RECs entails in contrast to clinical ethics consultation. But what seems undeniable to me is that there is some sort of expertise around ethics that is required in RECs, and for lack of a better term I am in favor of calling it ethics expertise, while clearly delineating it from moral expertise.

### Medical ethics versus philosophy

Given that there is some need of ethics expertise in RECs, or as it says in the statutes, a person with experience in the field of medical ethics, this raises another set of questions: who qualifies for this job? Which profession is it a part of? And where does the qualification stem from?

The interviewees were all quite unanimous in stating what their skillset included. How and where these skills were best acquired was seen in differing ways. Some of the participants viewed their skills as genuinely philosophical, while others thought of them as applied ethical, medical ethical, or research ethical. There was also dissent about whether or not applied ethical skills are also philosophical skills. Thus, while there seems to be some level of consensus on the kind of skills that ethics expertise entails, what is not clear is how and where to acquire this skillset and what profession it belongs to. However, it would seem to be vital to know this if one wants to reliably meet the need for ethics expertise mentioned above.

There are at least two different levels on which a possible explanation can be found—a theoretical one and a practical, or institutional, one. On a theoretical level the interviewees might disagree on the relationship between philosophy and applied ethics.^4^ There is some controversy in the literature as to what role moral theory plays in applied ethics. While some authors view applied ethics as a part of moral philosophy (e.g. LaFollette 2005; Archard and Lippert-Rasmussen 2013), others argue that applied ethics is merely concerned with the application of moral theory to concrete cases (cf. Caplan 1980; Kaminsky 2005). Based on this understanding, applied ethics can be seen as located outside of moral philosophy. But it does seem hard to imagine applied ethics being so separate from philosophy that the ethics part does not derive in some way from philosophy (assuming it also does not derive from theology) (cf. Flynn 2021). Arguably, any theories and principles that are used in medical ethics have their roots in moral philosophy. So, when some interviewees explicitly say that the crucial ethics knowledge in the committee is not philosophical knowledge, while others say of the same kind of knowledge that it is both philosophical and applied ethical, perhaps the disagreement is not exclusively located on the theoretical level. It might also be due to a diverging self-understanding on a practical, or institutional, level.

In fact, it might be necessary to differentiate between at least two academic disciplines called medical ethics, specifying one that is a sub-discipline of philosophy and one that is separate from philosophy. All of the interviewees are philosophers by training, but they work in different institutional settings. Generally, those who thought of their skills as applied ethical rather than as philosophical, or differentiated between the two at all, were the ones whose institutional home (as in workplace) was in medical ethics as opposed to in a philosophy department. So one possible reason why the interviewees have such different views regarding their profession or what discipline their knowledge/skills belong to, might be the differences in the institutional culture of their workplace. The interviews seem to suggest that this line could at least loosely be drawn between medical ethics departments and philosophy departments. This would explain why it is mainly those working in a medical ethics setting who view their knowledge as medical ethical knowledge, whereas those working in a more classical philosophical setting view the same kind of knowledge as philosophical knowledge. Methods and content may be similar and also formed by the shared experience of being a member in a standardised research ethics committee. But whether individuals understand themselves or the skills they are applying as applied ethical or philosophical might depend on the institutional culture of their workplace.^5^ Additionally, the different contexts in which medical ethics discourses take place—academic, clinical, and policy-oriented—might also lead to different understandings of the discipline (cf. Flynn 2021).

Gaining some clarity concerning the shared skills and where best to acquire them might be helpful in order to understand what potential expertise in ethics entails and who might have it and as a result of what training. In order to gain such clarity it might be interesting to conduct further research concerning the institutional culture and the views of both medical ethicists with a philosophical background and those with a medical background.

### Ethics and law

The previous paragraph has shown that if some of the philosophers in research ethics committees don’t view their ethical knowledge as philosophical knowledge, it is questionable where the ethics and hence the normativity comes from. Some of them have also claimed that what they do in the committee is mostly apply pre-existing principles and guidelines to concrete cases. Combined with the fact that some also find what they do in the committee to be very similar to what the legal experts do, this raises the question of how the ethical perspective can be meaningfully set apart from the legal perspective in the context of RECs.

Putting the question into more concrete terms, one could also ask: Is there an ethical sphere within the legal one? Research ethics committees can in a way also be described as law commissions. Their positive vote is required by law for clinical studies on medication and medical devices,^6^ making their statements administrative acts.^7^ Accordingly, all RECs require at least one person with a degree in law as per their statute. Considering the fact that not all statutes require the committee to have a person with an expertise in ethics, it seems that legal questions have a certain importance in the committee that ethical questions might not have.

Now, when philosophers in research ethics committees say that they are essentially doing the same job, or at least a similar job, as the legal expert, or that they have the same or similar competencies, this prompts the question of where possible differences lie. As we have seen, some interviewees stated that ethical questions are addressed in the committee after the legal questions have been settled (P 1), some say that ethical discussions take place in areas where legal regulations aren’t clear (P 6) and some say that what is done in RECs is mostly the interpretation of legal regulations (P 2). So not much can be said about the relationship, besides that it is very unclear. But the fact that it seems unclear and that the very people involved aren’t seeing eye to eye as to where ethical questions are to be located in relation to legal questions in a committee that is called an “ethics committee” but that is essentially also a law commission, is interesting in itself. In order to bring to shed more light onto this topic it might be interesting to look into it further, for example by conducting more empirical research, systematically analysing the questions that are being discussed in RECs and trying to sort them into ethical and legal questions and see where the two intersect. On a theoretical level, the question that seems most pressing is how ethics and law intersect in the interpretation of law. This, of course, touches on old discussions on legal positivism, but could be fruitful in light of this concrete field of application.

## Conclusion

The study indicates that members of research ethics committees (RECs) with a background in philosophy or applied ethics generally don’t view themselves as ethics experts, although it is not completely clear what understanding of expertise this assessment rests on. Members included in the study are rather unanimous about the competencies they think they contribute to the committee, but they disagree about whether those competencies are distinctly philosophical ones or applied ethical ones. There is also some controversy about how important these competencies are. Some members argue that their being a medical layperson is also an important, if not the most important, feature they contribute to the committee. Moreover, some members don’t see a big difference between their role and the role of the member with legal training.

The study also hints at three topics that should be explored further. Firstly, the study suggests a lack of consensus among REC-members about their interpretations of the concept of ethical expertise and about their own role within the commitees. Communication among REC-members about these topics should be intensified in order to develop a shared understanding. A generally accepted concept of ethics expertise that is in line with their specific role would certainly be helpful in further assessing the role of philosophers or ethicists in research ethics committees. For this purpose it would be helpful to distinguish between moral and ethics expertise. A good starting point for a concept of ethics expertise that is relevant to practice is Lisa Rasmussen’s concept of ethics expertise that is tailored for the context of clinical ethics consultation and will have to be adjusted in certain aspects for the context of RECs.

This in turn touches on the second topic: When thinking about ethics expertise or similar concepts, the question arises of where to gain said expertise. The interviews have shown that the relationship between philosophy and applied ethics as disciplines is somewhat unclear. The reason for this might be located on a theoretical level, or on a practical—institutional—level. Gaining more clarity concerning the shared skills and where best to acquire them would again be helpful in order to understand what a potential expertise in ethics entails and who might have it and due to what training.

Thirdly, in keeping with this line of thought, it is also important to clarify the relationship between ethics and law in this specific context. That this is necessary is apparent: the committees are called (research) ethics committees, yet are essentially also law commissions, so their status is unclear on this basis in any case, and the interviews have reinforced this by showing that it isn’t even always clear where the difference between the ethicist and the legal expert in the committee lies.

For future empirical research, a study comparing the different roles of ethics experts and the concomitant different concepts of ethics expertise remains a desideratum. It would be interesting to look at the philosopher’s role in HCECs, because these are less standardised and less constrained by laws, so it is likely that there is more actual ethics taking place and that their role is almost certainly very different than that of the philosophers in RECs.

## Data Availability

The original versions of the translated citations can be requested from the author.

## Footnotes

1 This kind of committee is also often called Human Research Ethics Committee (HREC).

2 Whether he or she is a current or a former member cannot be disclosed for reasons of anonymity.

3 The difference between philosophical and ethical expertise or knowledge was also the subject of the interviews, and will be addressed below.

4 Applied ethics meant here as a summary of the aforementioned categories: applied ethics, medical ethics, and research ethics.

5 My personal experience suggests that the culture in medical ethics departments is very different from the culture in philosophical departments, and more oriented towards medicine and the sciences, at least when it comes to publishing practices and general working styles. So the idea that the institutional culture also influences the self-image or the understanding of one’s discipline does not seem far-fetched.

6 *Arzneimittelgesetz* and *Medizinproduktegesetz.*

7 See Vöneky 2010, p. 596f.
